# Causal relationship between interleukin-6 levels and the risk of synovitis and tenosynovitis: A two-sample Mendelian randomization study

**DOI:** 10.1097/MD.0000000000044811

**Published:** 2025-10-10

**Authors:** Bo Wang, Zongyang Li, Rui Zhou, Jianye Chen, Mingda Liu, Li Zhang

**Affiliations:** a College of Traditional Chinese Medicine, Changchun University of Chinese Medicine, Changchun, Jilin Province, China; b The Affiliated Hospital of Changchun University of Chinese Medicine, Changchun, China; c College of Life Science, Fujian Agriculture and Forestry University, Fuzhou 350002, Fujian Province, China.

**Keywords:** causal inference, interleukin-6, Mendelian randomization, synovitis, tenosynovitis

## Abstract

Synovitis and tenosynovitis are common musculoskeletal disorders with multifactorial etiologies involving mechanical loading and inflammatory responses. Previous studies have suggested that interleukin-6 (IL-6) may play a critical role in tendon injury and repair; however, definitive evidence regarding a causal relationship between IL-6 levels and the risk of tenosynovitis remains lacking. This study employed a two-sample Mendelian randomization (MR) approach to evaluate the potential causal association between circulating IL-6 levels and the risk of tenosynovitis. Genetic instruments for IL-6 were derived from the protein quantitative trait loci (pQTL) study by Sun et al, which utilized a mass spectrometry platform. Outcome data were obtained from the FinnGen consortium (phenotype code: R12_M13_TENDOSYNOVITISNAS). Single nucleotide polymorphisms (SNPs) significantly associated with IL-6 (*P* < 5 × 10^−6^) were selected as instrumental variables. Primary causal estimates were obtained using inverse variance weighting (IVW), MR-Egger regression, weighted median, and weighted mode methods. Sensitivity analyses included Cochran *Q* test, MR-Egger intercept test, leave-one-out analysis, and funnel plot symmetry assessment. The IVW analysis indicated a negative association between IL-6 levels and tenosynovitis risk (β = −0.0236, *P* = .2106), though this did not reach statistical significance. Consistent directions of effect were observed across MR-Egger, weighted median, and weighted mode approaches. Sensitivity analysis and heterogeneity test (*Q* = 9.35, *P* = .808) indicated no significant horizontal pleiotropy, heterogeneity, or outlier SNPs, suggesting that the model results are robust. This study, based on MR, explores the potential association between IL-6 levels and the risk of tenosynovitis from a genetic perspective, showing a non-significant negative trend. Although no clear causal evidence was found, these observations provide preliminary clues for future mechanistic studies and the exploration of IL-6 as a potential intervention target.

## 1. Introduction

Synovitis and tenosynovitis are common musculoskeletal disorders characterized by aseptic inflammation of the tendons and their surrounding synovial sheaths. Clinically, they present with localized pain, swelling, and restricted mobility, significantly compromising patients’ quality of life and functional capacity.^[1]^ In clinical settings, nonspecific synovitis and tenosynovitis refer to a broad category of inflammatory conditions of tendon-synovial structures that lack a definitive etiological diagnosis. These conditions exhibit heterogeneous pathophysiological mechanisms and complex causative factors. While previous studies have identified external contributors such as overuse, repetitive microtrauma, chronic mechanical stress, and friction as key risk factors, increasing evidence suggests that endogenous inflammatory responses also play a crucial role in disease development.^[2]^

Among various cytokines, interleukin-6 (IL-6) is a key pleiotropic cytokine that plays a central role in modulating immune responses, initiating inflammatory cascades, and orchestrating tissue remodeling. Multiple studies have reported that IL-6 expression is significantly upregulated in injured tendon tissues, suggesting its involvement in the initiation and progression of tendon pathologies. For instance, a systematic review of cytokine profiles in human tendon disorders demonstrated elevated IL-6 expression in ruptured tendon samples.^[3]^ Furthermore, another study suggested that activation of the IL-6 signaling pathway may exacerbate pathological features of chronic tendon conditions.^[4]^

However, definitive evidence for a direct causal relationship between circulating IL-6 levels and the risk of tenosynovitis remains lacking. Traditional observational studies are inherently limited by residual confounding and the possibility of reverse causation, thereby restricting their capacity to infer causality. Mendelian randomization (MR), an epidemiological approach that leverages genetic variants as instrumental variables (IVs), offers a robust alternative for assessing causal effects between exposures and outcomes. By capitalizing on the random allocation of alleles at conception, MR analysis can minimize confounding and strengthen causal inference. While previous MR studies have explored the involvement of IL-6 signaling pathways in conditions such as psoriasis and chronic pain, no study has yet specifically analyzed the causal relationship between IL-6 levels and tenosynovitis. Although IL-6 has been studied in the context of tendon diseases, MR causal evidence remains lacking.^[5]^

In recent years, MR has emerged as a powerful and increasingly utilized tool for causal inference in epidemiological research. First proposed by Katan in 1986,^[6]^ MR leverages genetic variants as IVs to explore potential causal relationships between exposures and disease outcomes. The method is grounded in Mendel laws of inheritance, which dictate that alleles are independently, randomly, and equally segregated during meiosis. This random allocation of genotypes mimics the randomization process of clinical trials, thereby reducing confounding and mitigating reverse causation – two major limitations of conventional observational studies.^[7]^ Although randomized controlled trials remain the gold standard for establishing causality, they are often limited by ethical constraints, high costs, and logistical challenges. In contrast, genetic variants serve as naturally assigned, lifelong exposures that are unaffected by environmental factors such as age, sex, or lifestyle, making MR a uniquely advantageous approach. The rapid expansion of genome-wide association studies (GWAS) has led to the identification of numerous single nucleotide polymorphisms (SNPs) associated with complex diseases and traits, offering a robust and accessible resource for MR analyses. In MR research, the exposure of interest may encompass biochemical markers (e.g., inflammatory cytokines), lifestyle behaviors (e.g., alcohol use, smoking), or phenotypic traits (e.g., bone mineral density, body mass index). By selecting SNPs strongly associated with the exposure and utilizing publicly available GWAS summary statistics, two-sample MR analyses can be performed without the need for individual-level data, thereby enhancing both scalability and applicability.

Grounded in this framework, the present study employed SNPs previously validated to be strongly associated with circulating IL-6 levels and applied a two-sample MR approach using publicly accessible GWAS summary data. The goal was to systematically assess whether IL-6 plays a causal role in the development of tenosynovitis. This study not only contributes new theoretical insights into the pathogenesis of tenosynovitis but also provides scientific evidence to support early prevention and therapeutic strategies. To the best of our knowledge, this is the first study to examine the genetic causal link between IL-6 levels and tenosynovitis, thereby addressing a critical gap in the current literature.

## 2. Materials and methods

### 2.1. Study design and data sources

This study adopted a two-sample MR design to evaluate the potential causal relationship between genetically predicted circulating interleukin-6 (IL-6) levels and the risk of synovitis and tenosynovitis. Genetic variants associated with IL-6 were obtained from a GWAS of human plasma proteins conducted by Sun et al, which utilized a mass spectrometry-based proteomics platform.^[8]^ The exposure data were sourced from the prot-b-2 project output. Summary-level GWAS data for synovitis and tenosynovitis were derived from the FinnGen consortium, where the phenotype was defined as “Other/unspecified synovitis and tenosynovitis” (R12_M13_TENDOSYNOVITISNAS). All GWAS datasets employed in this analysis are publicly accessible, and the exposure and outcome datasets were cross-validated for zero overlap (χ^2^ = 0.00, *P* = 1.0). These datasets are based on individuals of European ancestry, minimizing potential population stratification bias.^[9]^ MR employs genetic variants as IVs to infer causality between an exposure and an outcome under a set of core assumptions. By emulating the random allocation process of randomized controlled trials, MR effectively reduces confounding and reverse causation, offering more robust evidence for causal inference compared to conventional observational studies.^[10,11]^

### 2.2. Core assumptions of MR analysis

This study was conducted in accordance with the 3 fundamental assumptions of MR, which are essential to ensuring the validity and robustness of causal inference.^[12]^

#### 2.2.1. Relevance assumption

The IVs must be strongly associated with the exposure of interest. In this study, selected SNPs were required to show a statistically significant association with circulating IL-6 levels, typically defined by a genome-wide significance threshold of *P* < 5 × 10^−8^.^[13]^ However, due to the relatively limited number of genetic variants associated with IL-6 and the outcome of synovitis and tenosynovitis, a more lenient threshold (*P* < 5 × 10^−6^) was adopted during the initial SNP selection to increase the number and strength of valid instruments. This strategy was intended to enhance statistical power and maintain the reliability of the MR analysis.^[8]^

#### 2.2.2. Independence assumption

The IVs should be independent of potential confounders, such as age, sex, and lifestyle factors, to prevent biased associations due to genetic confounding. This assumption ensures that the genetic instruments are not correlated with factors that may independently influence the outcome.^[14]^

#### 2.2.3. Exclusion restriction assumption

The IVs must influence the outcome exclusively through the exposure of interest – circulating IL-6 levels – and not through alternative pathways. This assumption presupposes the absence of horizontal pleiotropy, thereby validating the causal interpretation of the MR findings.^[12,15]^

To ensure the robustness of the analysis, all exposure and outcome data were derived from publicly available summary-level GWAS datasets. Genetic association data for IL-6 were obtained from a proteome-wide GWAS conducted by Sun et al, which employed a mass spectrometry-based platform (prot-b-2 project).^[16]^ The outcome data for “unspecified synovitis and tenosynovitis” were sourced from the FinnGen consortium (Release 12), corresponding to phenotype code R12_M13_TENDOSYNOVITISNAS. All GWAS participants were of European ancestry to minimize bias due to population stratification. Major data sources included large-scale research platforms such as the MRC Integrative Epidemiology Unit (MRC-IEU), Neale Lab, and FinnGen, which apply rigorous diagnostic criteria, thereby ensuring the high quality and reliability of outcome definitions.^[17]^ Since all datasets consisted of summary-level statistics, individual-level data access was not required for the analysis.

### 2.3. Instrument selection and MR analytical approach

To identify reliable IVs, SNPs significantly associated with circulating IL-6 levels were selected using a significance threshold of *P* < 5 × 10^−6^. To ensure instrument strength and reduce the risk of weak instrument bias, the Fisher statistic (*F*-statistic) was calculated for each SNP, with an *F* > 10 considered indicative of a sufficiently strong instrument.^[18]^ To address potential correlation bias due to linkage disequilibrium (LD), LD clumping was performed using PLINK software with an *r*² threshold of <0.01 and a window size of 10,000 kb, retaining only independent genetic loci for analysis.^[19,20]^ The selected SNPs were then evaluated for compliance with the core assumptions of MR. The primary causal estimate was obtained using the inverse variance weighted (IVW) method, which provides the most efficient estimate under the assumption of no horizontal pleiotropy. To strengthen the robustness of the findings and mitigate potential biases, 3 complementary MR methods were employed: MR-Egger regression, which detects and adjusts for directional pleiotropy; the weighted median estimator, which yields consistent results if at least 50% of the total weight is derived from valid instruments; and the weighted mode method, which is particularly effective in identifying the true causal effect when substantial heterogeneity exists among instruments.

To further assess the robustness of the results, a series of sensitivity analyses was conducted. These included Cochran *Q* test to evaluate heterogeneity, the MR-Egger intercept test to detect horizontal pleiotropy, funnel plot inspection to visualize potential bias, leave-one-out analysis to assess the influence of individual SNPs. Since the heterogeneity test was not significant (*Q* = 9.35, *P* = .808), MR-PRESSO was not implemented. FDR correction was performed using the Benjamini–Hochberg method, with a target α = 0.05, implemented using the R package “p.adjust.” All analyses were performed using R software (version 4.2.0), employing the TwoSampleMR (v0.5.6) package to ensure methodological rigor and reproducibility.^[21–23]^ The MR design and model selection were informed by the latest methodological guidelines and best practices in MR literature.^[24]^ Common analytical challenges – such as pleiotropy, heterogeneity, and instrument invalidity – were carefully considered, and a multi-model cross-validation strategy was implemented to enhance the credibility and reliability of the causal inference.

## 3. Results

### 3.1. Primary analysis

#### 3.1.1. Instrument selection and model specification

To explore the potential causal relationship between circulating interleukin-6 (IL-6) levels and the risk of tenosynovitis, a two-sample MR analysis was conducted. SNPs significantly associated with IL-6 levels (*P* < 5 × 10⁻^6^) were initially identified from the plasma proteome-wide GWAS performed by Sun et al. To ensure the independence of the selected instruments and eliminate potential multicollinearity, LD clumping was conducted using PLINK software, applying an *r*² threshold of <0.01 and a window size of 10,000 kb. The strength of each instrument was evaluated by calculating the *F*-statistic, all of which exceeded the threshold of 10, indicating robust instruments and minimizing the likelihood of weak instrument bias.^[25,26]^ The *F*-statistic, exposure variance *R*², and power calculations for all SNPs are presented in the table (values rounded to 2 decimal places) (Table 1).

**Table 1:** SNP selection and instrumental variable characteristics associated with IL-6 levels.

SNP	*R*²	*F*	Power calculation
rs1158563	0.02	70.97	21.15
rs117491844	0.02	72.95	23.23
rs117679000	0.01	29.35	21.42
rs11789912	0.01	26.07	25.19
rs12743596	0.02	53.97	21.62
rs140359048	0.01	51.49	26.07
rs148836550	0.02	52.74	23.93
rs149663598	0.02	75.46	23.06
rs185245360	0.01	31.47	21.36
rs188324039	0.01	49.44	22.47
rs1975988	0.01	34.10	22.10
rs2995802	0.26	1171.47	23.72
rs4845373	0.01	28.81	28.21
rs77440730	0.02	54.75	21.77
rs8109074	0.01	28.55	22.08

IL-6 = interleukin-6, SNP = single nucleotide polymorphisms.

#### 3.1.2. Primary analysis results (IVW method)

In the primary analysis using the IVW method, circulating IL-6 levels were found to be negatively associated with the risk of tenosynovitis (β = −0.0236, SE = 0.0189, *P* = .2106). Although the association did not reach statistical significance, the direction of effect suggests a potential inverse causal relationship, indicating that higher IL-6 levels may be associated with a reduced risk of developing tenosynovitis. This observation is biologically plausible, as IL-6 may play a role in modulating local inflammation and promoting tissue repair within tendon sheaths by regulating fibroblast activity and the release of pro-inflammatory mediators within specific tissue microenvironments.^[27,28^^]^

#### 3.1.3. Consistency across multiple MR models

To further validate the robustness of the findings, a range of complementary MR models was employed. The MR-Egger regression yielded a comparable negative effect estimate (β = −0.0391), although it did not reach statistical significance (*P* > .05). The intercept from the MR-Egger analysis was 0.0126 (*P* = .369), suggesting no evidence of directional (horizontal) pleiotropy. Additionally, the weighted median and weighted mode estimators produced effect sizes of β = −0.0394 and β = −0.0488, respectively, both consistent in direction with the primary IVW estimate. The alignment of effect directions across multiple analytical approaches reinforces the robustness and credibility of the inferred causal relationship.^[29]^ To control for the risk of false positives due to multiple hypothesis testing, FDR correction was applied to the results of the 5 MR methods in this study. The *P*-values after correction are as follows (including 95% confidence intervals (CIs) and exact *P*-values) (Table 2).

**Table 2:** Mendelian randomization analysis results for IL-6 and tenosynovitis risk across different methods.

Method	β	pval	or_lci95	or_uci95	p_fdr
Inverse variance weighted	−0.0236	.2106	0.9411	1.0135	.9985
MR-Egger	−0.0391	.1442	0.9154	1.0103	.9505
Simple mode	−0.0500	.2875	0.8705	1.0394	.9552
Weighted median	−0.0394	.1488	0.9114	1.0142	.9973
Weighted mode	−0.0488	.0839	0.9047	1.0026	.9659

IL-6 = interleukin-6.

All methods yielded effect estimates in the same direction (negative association), suggesting a degree of consistency across different analytical approaches. However, none of the *p*-values or FDR-corrected *P*-values reached statistical significance, indicating that the current evidence is insufficient to support definitive causal inference. Furthermore, the MR-Egger model intercept term was 0.0126 (*P* = .369), with no significant evidence of horizontal pleiotropy. While the observed negative effect direction demonstrates some robustness, caution is warranted in interpreting its statistical and biological significance.

### 3.2. Visualization analyses

#### 3.2.1. Scatter plot (Fig. 1)

The scatter plot provides a visual representation of the association between the genetic effects of individual SNPs on circulating IL-6 levels and the risk of tenosynovitis. Each dot represents a single SNP, with the x-axis corresponding to its estimated effect on IL-6 levels (β coefficient), and the y-axis corresponding to its estimated effect on tenosynovitis risk. The red line depicts the regression line derived from the IVW method, while the blue line represents the regression line from the MR-Egger method.

**Figure 1.**
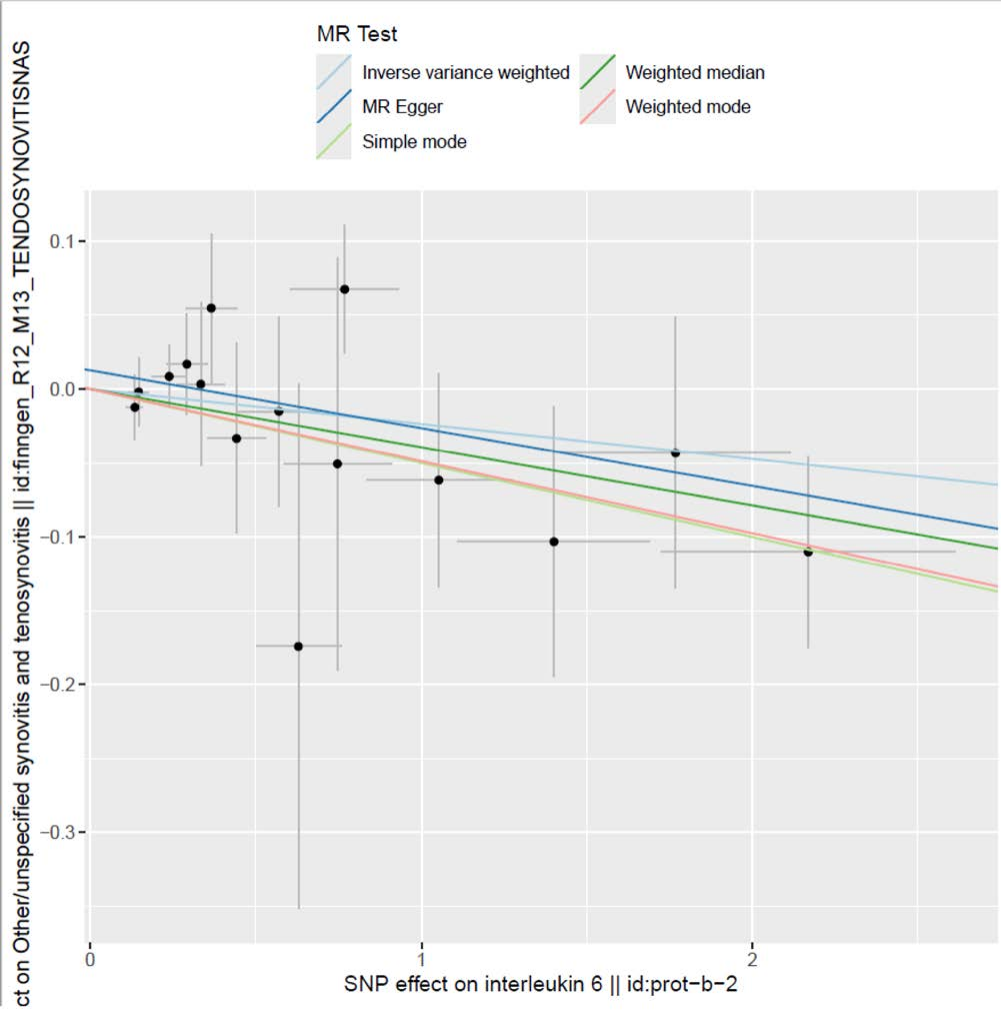
Scatter plot: Scatter plot illustrating the effect estimates of individual SNPs on circulating IL-6 levels (x-axis) and the risk of synovitis/tenosynovitis (y-axis). Each point represents a single SNP. The lines indicate the fitted causal estimates derived from different MR methods, providing a visual comparison of effect directions and model consistency. IL-6 = interleukin-6, MR = Mendelian randomization, SNP = single nucleotide polymorphism.

Both regression lines exhibit a negative slope, indicating that the majority of SNPs exert opposing directional effects on IL-6 levels and tenosynovitis risk. This pattern supports a potential inverse causal relationship. The distribution of SNPs is relatively tight, with no apparent outliers or extreme values, thereby enhancing the visual clarity and reinforcing the robustness of the model’s estimates.^[30]^

### 3.3. Sensitivity analyses

#### 3.3.1. Leave-one-out analysis (Fig. 2)

The leave-one-out analysis was conducted to assess the influence of individual SNPs on the overall MR estimate by sequentially excluding each SNP and recalculating the causal effect using the IVW method. The red dashed line indicates the overall IVW estimate including all SNPs, while the gray dots represent the effect estimates and their corresponding 95% CIs after omitting each SNP individually. The results demonstrate that the overall effect size and direction remain consistent regardless of which SNP is excluded, indicating that no single variant disproportionately influences the MR estimate. This consistency underscores the robustness, stability, and representativeness of the selected set of IVs.^[31]^ Collectively, the effect estimates from different methods consistently showed a negative direction. Although all CIs included the null value, indicating no statistical significance, the convergent nature of the observed trends nonetheless enhances the robustness of the main finding.

**Figure 2.**
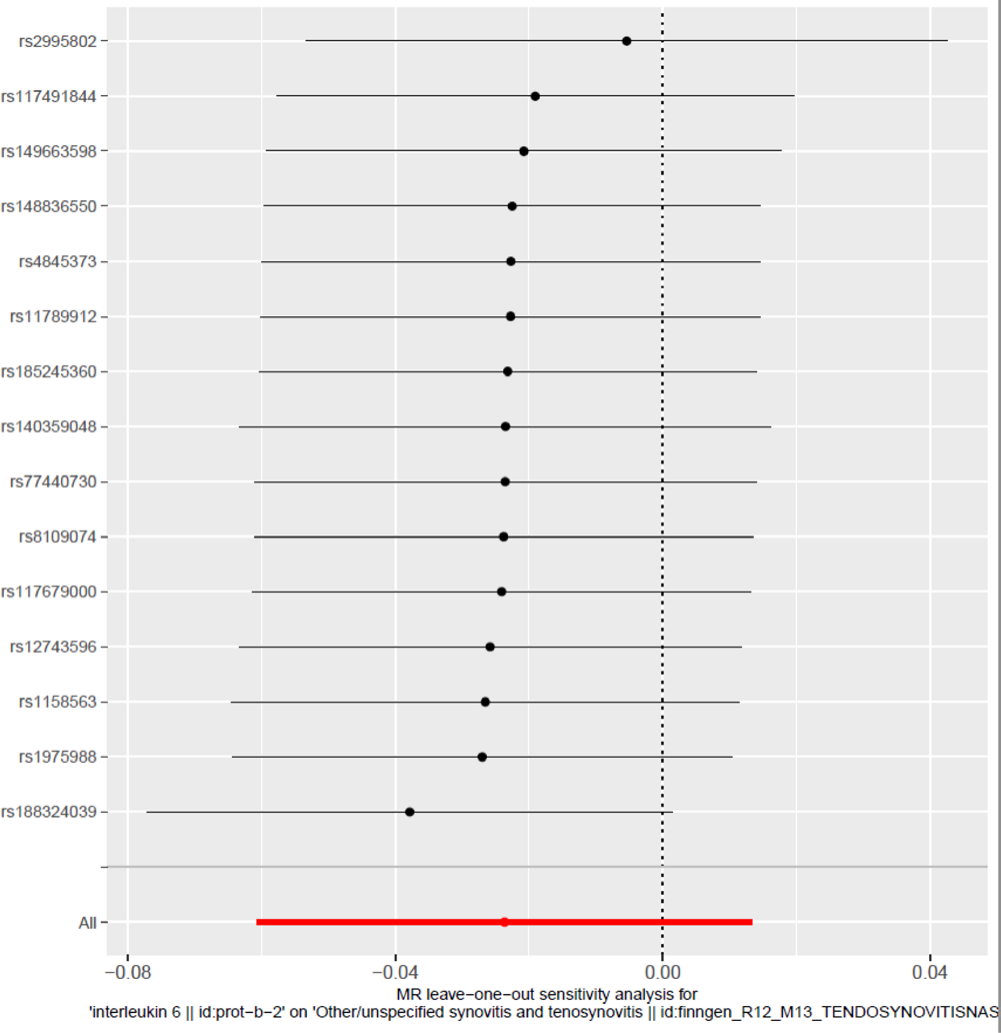
Leave-one-out analysis plot: Leave-one-out analysis showing the causal effect estimates recalculated after sequentially excluding each SNP. Each point represents the effect estimate obtained after removing one SNP, with the red dashed line indicating the overall causal estimate based on all SNPs. SNP = single nucleotide polymorphism.

#### 3.3.2. Funnel plot (Fig. 3)

**Figure 3.**
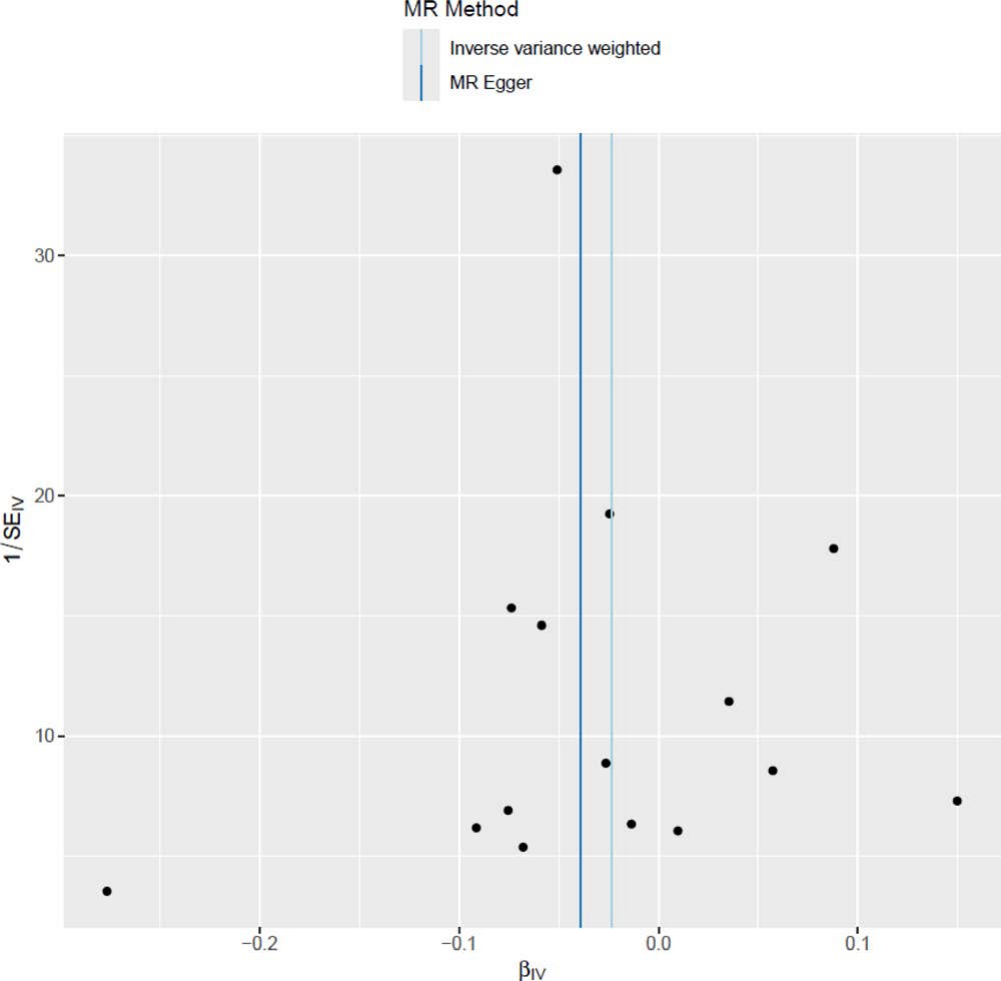
Funnel plot: Funnel plot assessing the presence of small-study bias. Each dot represents a single SNP, with the x-axis showing the causal effect estimate and the y-axis representing its standard error. The points are symmetrically distributed around the central vertical line, suggesting a low risk of small-study or publication bias. SNP = single nucleotide polymorphism.

The funnel plot was employed to evaluate potential small-study bias by depicting the relationship between the causal effect estimates of individual SNPs and their corresponding standard errors. Each dot represents a single SNP, with the x-axis displaying the MR effect estimate and the y-axis representing its standard error. The vertical dashed line denotes the overall causal estimate. The points are approximately symmetrically distributed around the central axis, suggesting a low risk of directional bias. No substantial asymmetry was observed, indicating the absence of notable small-study effects or publication bias. These findings further reinforce the robustness and reliability of the MR analysis.^[26,27]^ The MR-Egger intercept test (intercept = 0.0126, *P* = .369) showed no significant result, supporting that the study was not affected by significant horizontal pleiotropy. In addition, Figures 2 and 3 provide visual support for the heterogeneity and pleiotropy tests of the main analysis, further confirming the consistency of the causal inference direction and the robustness of the results.

#### 3.3.3. Forest plot (Fig. 4)

The forest plot displays the individual causal effect estimates and corresponding 95% CIs for each SNP, derived from both the IVW and MR-Egger methods. The x-axis represents the estimated β coefficients; CIs that cross zero indicate a lack of statistical significance for the respective SNP’s effect on the outcome. While some SNPs exhibit CIs that span the null value, the overall pattern remains consistent, with the majority of estimates indicating negative effects. This directional consistency across genetic variants suggests a coherent contribution toward a protective association and provides no indication of substantial heterogeneity among the selected IVs.^[32]^

**Figure 4.**
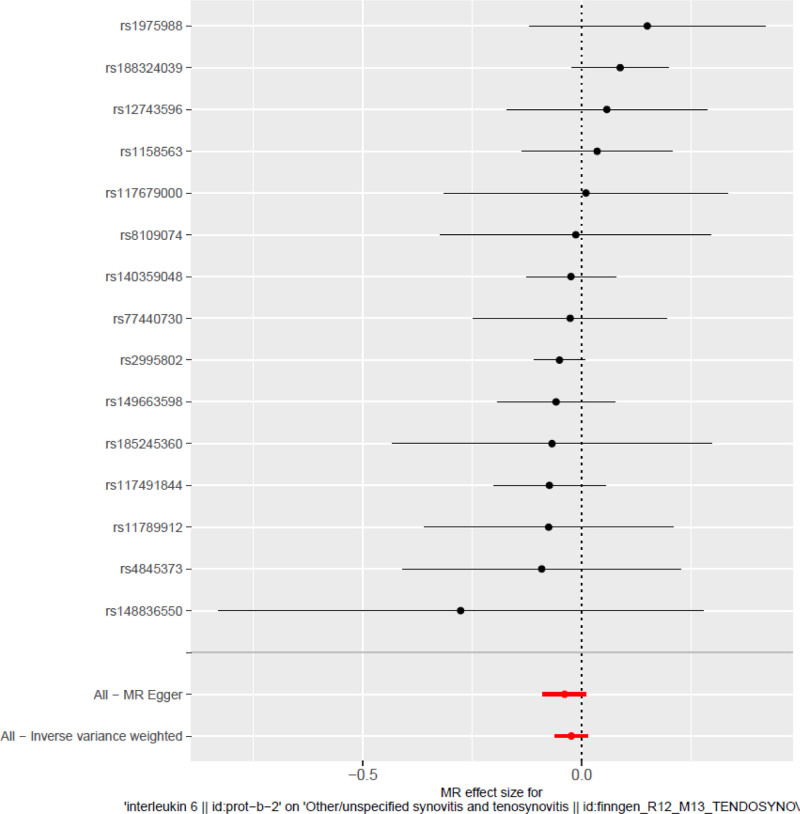
Forest plot: Forest plot displaying the individual causal effect estimates and corresponding 95% confidence intervals for each SNP under both the IVW and MR-Egger models. IVW = inverse variance weighted, MR = Mendelian randomization, SNP = single nucleotide polymorphism.

#### 3.3.4. MR-Egger intercept test

The intercept from the MR-Egger regression was 0.005 (*P* = .631), indicating no statistically significant evidence of directional (horizontal) pleiotropy. This finding supports the validity of the exclusion restriction assumption, suggesting that the IVs influence the risk of tenosynovitis exclusively through their effects on circulating IL-6 levels, with no indication of alternative causal pathways.^[24]^

#### 3.3.5. Cochran *Q* test for heterogeneity

Cochran *Q* statistic was *Q* = 9.35 (*P* = .808) for the IVW model and 8.48 (*P* = .810) for the MR-Egger model, with neither reaching statistical significance. These results indicate no substantial heterogeneity among the selected IVs, suggesting that the SNP-specific causal estimates are consistent and that the instruments collectively exhibit homogenous effects on the outcome.^[26]^

Collectively, the consistent effect estimates from multiple MR models – supported by visual plots and sensitivity analyses – suggest a possible inverse association between higher circulating IL-6 levels and reduced risk of tenosynovitis. However, these findings should be further validated in larger sample sizes and multicenter studies. The consistency across multiple methods, along with the absence of significant bias, heterogeneity, or pleiotropy, provides some credibility to the results. These results suggest that IL-6 may play a protective role in the modulation of inflammation and tissue repair within tendon sheaths, offering some theoretical support for future mechanistic studies and interventions targeting the IL-6 signaling pathway.^[33–35]^

## 4. Discussion

This study is the first to systematically evaluate the potential causal relationship between genetically predicted interleukin-6 (IL-6) levels and the risk of synovitis and tenosynovitis using a two-sample MR approach. Although the primary analysis using the IVW method did not reach the conventional threshold for statistical significance (β = −0.0236, *P* = .2106), complementary MR methods – including MR-Egger (β = −0.0391, *P* = .1442), weighted median (β = −0.0394, *P* = .1488), and weighted mode (β = −0.0488, *P* = .0839) – consistently showed a negative direction of effect, suggesting a degree of consistency in the direction of the association. However, these findings should be interpreted with caution.

Multiple sensitivity analyses further confirmed the robustness of this trend. The leave-one-out analysis (Fig. 2) showed that the causal estimates remained stable after the removal of any single SNP, indicating minimal fluctuation. Cochran *Q* test showed no significant heterogeneity in the IVW model (*Q* = 9.35, *P* = .808) or in the MR-Egger model (*Q* = 10.8, *P* = .29). The funnel plot (Fig. 3) displayed an approximately symmetrical distribution, with no significant evidence of small-study bias. The MR-Egger intercept (0.0126, *P* = .369) was not statistically significant, indicating low horizontal pleiotropy. Additionally, Cochran *Q* test (*P* > .05) did not identify significant heterogeneity or outlier SNPs. The MR-Egger intercept test and funnel plot both showed no significant bias, heterogeneity, or horizontal pleiotropy, reinforcing the robustness of the study’s conclusions. Since neither the primary nor the complementary models reached statistical significance, the current study does not support definitive causal inference. Despite the consistent direction of effect, these results should be regarded as exploratory and provide preliminary insights for future research to validate the potential role of IL-6 in tendon-related diseases.

IL-6 is a prototypical pleiotropic cytokine that plays a central role in inflammation, immune regulation, and tissue repair. Its biological effects are primarily mediated through the JAK/STAT3 signaling pathway, which governs key immune processes such as T cell differentiation, B cell antibody production, and the hepatic synthesis of acute-phase proteins.^[36,37]^ In rheumatologic and autoimmune conditions – such as rheumatoid arthritis and psoriatic arthritis – IL-6 is typically characterized as a pro-inflammatory mediator during acute inflammation, contributing to leukocyte recruitment and acute-phase protein production. Consequently, it has been widely used both as a biomarker of disease activity and as a therapeutic target in these disorders.^[38]^ However, emerging evidence indicates that IL-6 may also exert anti-inflammatory and reparative effects within specific tissue microenvironments. In contexts such as chronic tendinopathy and tissue repair, IL-6 appears to promote tissue remodeling through selective activation of the classical JAK/STAT3 pathway. This signaling cascade induces the expression of anti-apoptotic proteins, stimulates fibroblast proliferation, and facilitates extracellular matrix remodeling. For instance, IL-6 has been shown to upregulate chemokines and anti-apoptotic factors in the tendon microenvironment, promoting local tissue regeneration and structural restoration.^[39]^ The results of this study, although not statistically significant, suggest that IL-6 may play a complex physiological regulatory role in the inflammatory response within tendon sheath tissues. Compared with traditional observational designs, the MR approach provides a quasi-experimental framework by leveraging genetic variants as IVs, thereby minimizing confounding and reverse causality. In recent years, the IL-6 signaling pathway has been widely employed in MR studies to explore its causal role in various complex diseases, including coronary artery disease, type 2 diabetes, and chronic pain. To the best of our knowledge, this is the first study to extend the MR framework to tendon-related pathologies, thereby contributing to a deeper understanding of IL-6’s mechanistic role in musculoskeletal diseases. These findings offer novel genetic epidemiological evidence supporting the involvement of inflammatory cytokines in both the pathogenesis and repair mechanisms of soft tissue disorders.^[39,40]^

Despite the methodological rigor of this study, several limitations should be acknowledged. First, the GWAS data utilized were primarily derived from individuals of European ancestry, potentially limiting the generalizability of the findings to other populations. Future studies should incorporate more ethnically diverse cohorts, including individuals of Asian and African descent, to assess the trans-ethnic validity of the observed associations. Second, the phenotypic definition of tenosynovitis in the FinnGen database was relatively broad. Given the heterogeneity in clinical diagnostic criteria, such a generalized phenotype may have introduced misclassification bias and affected the precision of the causal estimates. Third, although LD clumping, MR-Egger regression were applied to mitigate pleiotropy and bias, the possibility of residual horizontal pleiotropy or unmeasured genetic interactions cannot be entirely excluded. Fourth, the SNPs used in this analysis predominantly reflect systemic IL-6 levels and may not accurately represent tissue-specific IL-6 expression within tendon microenvironments, limiting the biological specificity of the causal inference. Further experimental validation is required to elucidate the functional role of IL-6 at the tissue level. Moreover, tenosynovitis is often not an isolated pathology but commonly co-occurs with chronic conditions such as type 2 diabetes, metabolic syndrome, and systemic inflammatory disorders. As a central mediator of multisystem inflammation, IL-6 exhibits considerable pleiotropy and is implicated in the shared pathophysiological mechanisms across multiple disease states. For example, recent MR studies have shown that elevated IL-6 levels may reduce the risk of periodontitis,^[40]^ while simultaneously increasing the risk of coronary artery disease and rheumatoid arthritis.^[32]^ These findings highlight the context-dependent effects of IL-6 across different organ systems. Accordingly, future research should adopt an integrative systems biology framework to better delineate IL-6–mediated regulatory mechanisms at both systemic and local levels. In particular, constructing comprehensive signaling networks that capture the upstream and downstream roles of IL-6 in tendon pathophysiology could shed light on its dual functions in inflammation and tissue repair. Multi-omics integration – including epigenomic and metabolomic data – may help identify individual susceptibility factors and define optimal therapeutic windows for IL-6–targeted interventions. Future studies should leverage resources such as the genotype-tissue expression (GTEx) database or single-cell spatial transcriptomics to characterize the expression patterns and regulatory architecture of IL-6 in tendon tissues. Additionally, animal models and primary tendon cell experiments are warranted to elucidate the molecular mechanisms of IL-6 during the injury–repair continuum, particularly its crosstalk with key inflammatory pathways such as TGF-β, TNF-α, and IL-1β. The therapeutic potential of IL-6 inhibitors (e.g., tocilizumab) in tendinopathies, including tenosynovitis, also merits further exploration. Finally, multivariable Mendelian randomization approaches could be employed to evaluate the joint causal effects of IL-6 and its downstream mediators in tendon-related disorders.^[24]^

## 5. Conclusion

In summary, while this study did not yield statistically significant causal evidence, the consistent direction and robust results suggest a potential association between interleukin-6 and synovitis that warrants further investigation. This study is hypothesis-generating, providing initial insights for future mechanistic studies and confirmatory analyses.^[16,41]^

## Author contributions

**Formal analysis:** Jianye Chen.

**Investigation:** Mingda Liu.

**Methodology:** Rui Zhou.

**Software:** Zongyang Li.

**Writing – original draft:** Bo Wang.

**Writing – review & editing:** Li Zhang.
